# Report of the Committee on Publication

**Published:** 1867-07

**Authors:** 


Dr. N. S. Davis, Permanent Secretary, in behalf of the Committee of Publication, presented the following report:—

When the undersigned commenced his term of office as Secretary, more than ten years since, the treasury of this Society was considerably in debt for the publication of the Transactions of the preceding year. And the receipts into the treasury have not been sufficient to liquidate the indebtedness and pay the full current expenses of publication. Hence, three alternatives have recurred to your committee at each returning year, namely:—first, to omit the publication of the Transactions annually, for want of funds; second, to publish on the individual credit of the committee, with an annually increasing balance against the treasury, which by this time would have amounted to not less than $1000; or, third, to reduce the cost of publication to the amount actually in the treasury, or nearly so. The only possible mode of accomplishing the latter alternative was, to first print the record of proceedings, reports, and papers constituting the Transactions, in the medical periodical under the control of the Secretary, and furnish extra sheets enough to make up the required number of volumes of Transactions for the use of the Society, at the cost of simple presswork, paper, and binding. In this way, from $100 to $150 could be saved to the treasury annually.

Regarding the publication of the reports and papers read before the Society, in the medical periodicals as, in itself, a benefit, by giving them a wider circulatien in the profession, your committee did not hesitate to adopt the third alternative named. This course met the uniform approval of the Society until the meeting in Decatur, June, 1867. At that meeting, which is the only one that your Secretary has failed to attend during his term of office, this subject was referred to a committee, whose report stated that it was *not* desirable to have the Transactions published in the medical journals, and recommended that 100 copies be published, independently, for the members of the Society. This report was laid on the table, without any action by the Society. Hence, the Committee of Publication were left without either an approval of their past work, or any instructions for the future.

As soon after the last meeting as the material for the Transactions had been placed in the hands of the committee, it was found that the independent publication of 100 copies of the Transactions would cost at least $260, while there was, at that time, a balance of only $100 in the treasury. In this dilemma, your committee resorted to the same practice as in preceding years, by which they reduced the cost of publication $111, distributed copies of the Transactions to all members who had paid the annual assessment early in November, and left the treasury free from embarassment. It is to be hoped that the increased number of members in attendance on the present annual meeting will so increase the receipts of the Treasurer, that the

Transactions of this meeting can be immediately sent to press, as an independent publication. But if, as has heretofore been the case, the amount should be found insufficient, your publication committee should not be left in the embarassing position of having neither the positive approval nor the instructions of the Society.

Immediately after the last annual meeting, the full record of proceedings was furnished for publication in both the medical journals published in our State. Subsequently, written notices were sent by the Secretary to the chairman of each committee charged with the duty of making a report at the next annual meeting. Certificates of appointment were also sent to each delegate appointed to attend the meeting of the American Medical Association for 1867.

As the duties devolving on the Publication Committee depend, for their execution, mainly upon the Permanent Secretary, the undersigned would respectfully request the Society to appoint his successor whenever they deem it advisable to do so.

Respectfully submitted, in behalf of the Com. of Publication.

N. S. DAVIS, *Permanent Secretary.*

The report was accepted, and a vote of thanks unanimously tendered to Dr. Davis, for his services in superintending the publication of the Transactions of the Society.

Dr. J. H. Hollister presented the annual report of the Treasurer, showing a balance of $2 or $3 in the treasury.

The report was accepted and referred to the Auditing Committee.

On motion, the Society adjourned to 2 o'clock P.M.

AFTERNOON SESSION—SECOND DAY.

The meeting was called to order at 2 o'clock P.M., Dr. S. W. Noble in the chair.

Dr. D. Prince, Chairman of the Judicial Committee, made the following report, which was accepted and the committee discharged:—

*Whereas*, The claim of Dr. Addison Niles to membership in the Illinois State Medical Society rests upon the fact of his having been received as a delegate from a society which the Illinois State Medical Society has declared not entitled to representation; therefore,

*Resolved*, That the charges are not regularly before the Society.

The committee would further recommend, that inasmuch as the Quincy Medical Society was not heard in its defence last year, that the question of its right to representation be referred to a new committee, with instructions to report at the next meeting of this Society.

DAVID PRINCE,

DeLASKIE MILLER,

H. A. JOHNSON,

E. POWELL,

E. W. MOORE,

} *Committee.*

Dr. J. Robbins, of Quincy, offered the following as an amendment to the report:—

*Whereas*, The claim of Dr. Addison Niles to membership in this Society rests alone on the fact that he was delegated thereto by a body (the so-called Quincy Medical Society) which this Society has declared not to be entitled to representation; therefore,

*Resolved*, That the name of Dr. Addison Niles be stricken from the roll of members.

On motion of Dr. H. Noble, the amendment was laid on the table. The report of the Committee was adopted.

